# Modelling coronary flow after the Norwood operation: Influence of a suggested novel technique for coronary transfer

**DOI:** 10.21542/gcsp.2018.7

**Published:** 2018-03-14

**Authors:** Mohammed K. Al-Jughiman, Maryam A. Al-Omair

**Affiliations:** 1 Prince Sultan Cardiac Center, Al-Ahsa, Saudi Arabia; 2 King Faisal University, Al-Ahsa, Saudi Arabia

## Abstract

**Background:** The dynamic behavior of the aortic sinuses has an important function in the specific characteristics of coronary blood flow. Several publications have confirmed suboptimal myocardial perfusion after the Norwood procedure. Our study was undertaken to confirm four hypotheses. First, we hypothesized that there is more resistance to coronary flow due to coronary attachments to hypoplastic aortic root and sinuses. Also, as the amalgamation of the ascending aorta with the pulmonary artery occurs above the aortic root, the coronary blood flow is not fully in antegrade pattern. Second, performing the Norwood with our modification i.e., coronary transfer to the well-developed sinuses of the pulmonary root will result in less resistance to flow and a full antegrade flow pattern. This may eventually improve the long term ventricular and survival outcomes. Third, our modification is applicable to all procedures where the pulmonary root supplies the systemic circulation e.g., Norwood, Damus–Kaye–Stansel (DKS), and Yasui operations, whether applied to single or biventricular repair. Fourth, with our modification, the effect of the type of shunt; Sano vs. Blalock Taussig (BT shunt) on the coronary flow after the Norwood will be mitigated. This will give the surgeon more freedom to which shunt to use, and may make the surgeon keener to perform the BT shunt in order to avoid the ventricular scar associated with the Sano shunt which will negatively impact the ventricular function.

**Methods:** Computational fluid dynamic (CFD) simulations were performed to evaluate flow streamlines and to quantify flow distribution and total pressure drop in the coronary branches in both Norwood (pre-transfer) and modified Norwood (post-transfer) models. Comparisons between the two models were performed.

**Results:** The systolic flow rate in all coronary branches was higher in the post-transfer model in the proportions of: left main 5%, left anterior descending (LAD) 6%, left circumflex (LCx) 3.5%, and right coronary artery (RCA) 7.2% higher flow rates. In diastole, pressure drop from the aortic inlet to distal left main and distal right main was substantially less in the post-transfer model.

**Conclusion:** Post-transfer model has produced more favorable coronary hemodynamics in all coronary branches. As a result, performing our modification could potentially improve the long term ventricular and survival outcomes.

## Background

The dynamic behavior of the aortic sinuses has an important function in the specific characteristics of coronary blood flow. Several publications^1,2^ have confirmed the suboptimal myocardial perfusion after Norwood procedure. Our study was undertaken to confirm four hypotheses. First, we hypothesized that there is more resistance to coronary flow due to coronary attachments to hypoplastic aortic root and sinuses. Also, as the amalgamation of the ascending aorta with the pulmonary artery occurs above the aortic root, the coronary blood flow is not fully in antegrade pattern. Second, performing the Norwood with our modification i.e., coronary transfer to the well-developed sinuses of the pulmonary root will result in less resistance to flow and a full antegrade flow pattern. This eventually will improve the long term ventricular and survival outcomes. Third, our modification is applicable to all procedures where pulmonary root supplies the systemic circulation e.g., Norwood, DKS, and Yasui operations whether applied to single or biventricular repair. Fourth, with our modification, the effect of the type of shunt (Sano vs. BT shunt) on the coronary flow after the Norwood procedure will be mitigated. This will give the surgeon more freedom to which shunt to use, and may make the surgeon keener to perform BT shunt in order to avoid the ventricular scar associated with Sano shunt which will negatively impact the ventricular function.

## Methods

### DICOM to STL conversion: turning an MRI into a 3-D digital model

**Figure 1.**
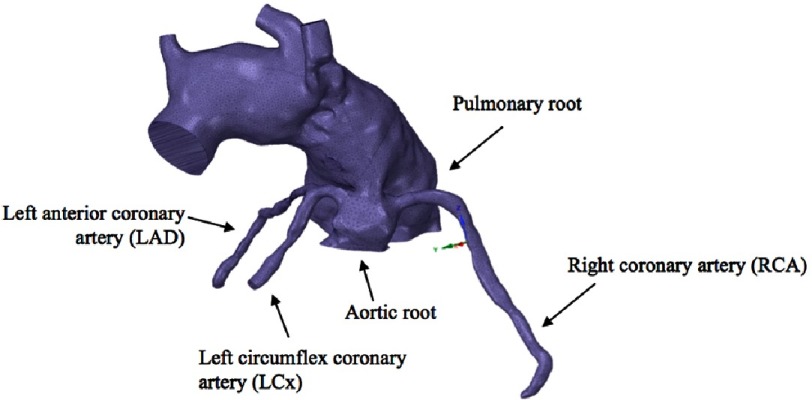
Images from an MRI scan were converted into a 3-D STL CAD model.

A patient-specific MRI post Norwood procedure for hypoplastic left heart syndrome was used to convert a Digital Imaging and Communications in Medicine (DICOM) format into a 3-D geometry. This 3-D geometry was exported in a Stereolithographic (STL) computer-aided design (CAD) format which can be imported into the ANSYS CFD Software (Figure 1).

### Final 3-D model in STL format: preparing for computational modeling

**Figure 2.**
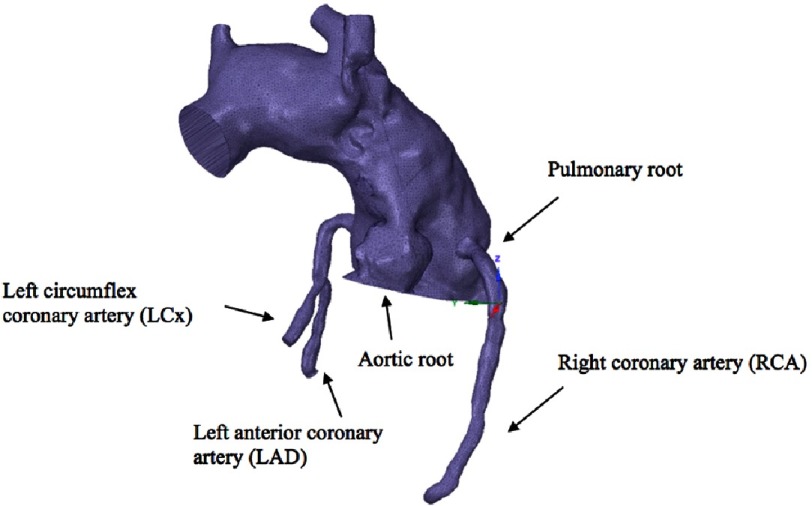
3-D CAD post coronary transfer to the pulmonary root model.

**Figure 3.**
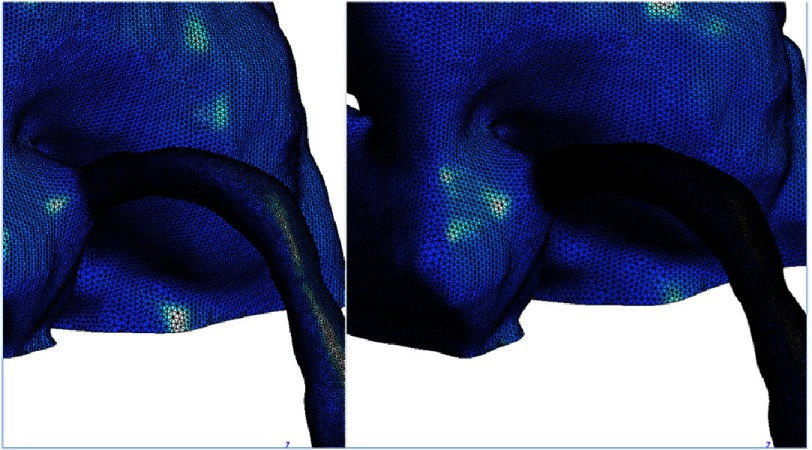
Mesh created on the original Norwood ‘pre-transfer’ geometry. Two mesh densities to check mesh insensitivity were created: coarse (left) and fine (right).

**Figure 4.**
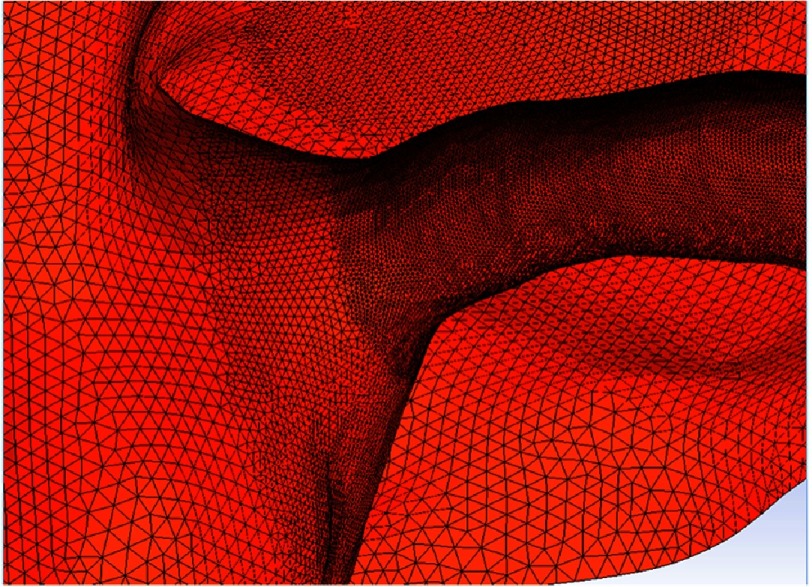
Close-up of the CFD mesh entering the right coronary artery. The mesh is refined in the coronaries to resolve their narrower lumens.

In our modification, the right coronary artery button is transferred and attached to the right pulmonary sinus, and the left main coronary button is transferred and attached to the left pulmonary sinus (Figure 2). Mesh was created on the original Norwood geometry. Two mesh densities were developed to check mesh insensitivity: coarse and fine meshes (Figure 3). Mesh density was increased in the coronaries to resolve their narrower lumens (Figure 4). The mesh was imported into ANSYS Fluent for setup and solving. The simulations were setup as steady state using Fluent’s Pressure-Based solver and Absolute Velocity formulation.

**Figure 5.**
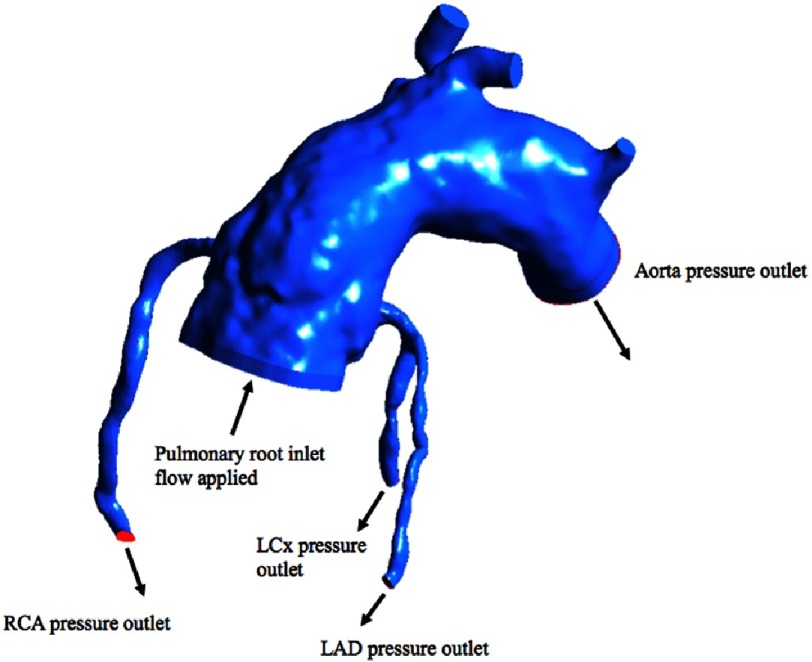
Modal evaluation in systole.

**Figure 6.**
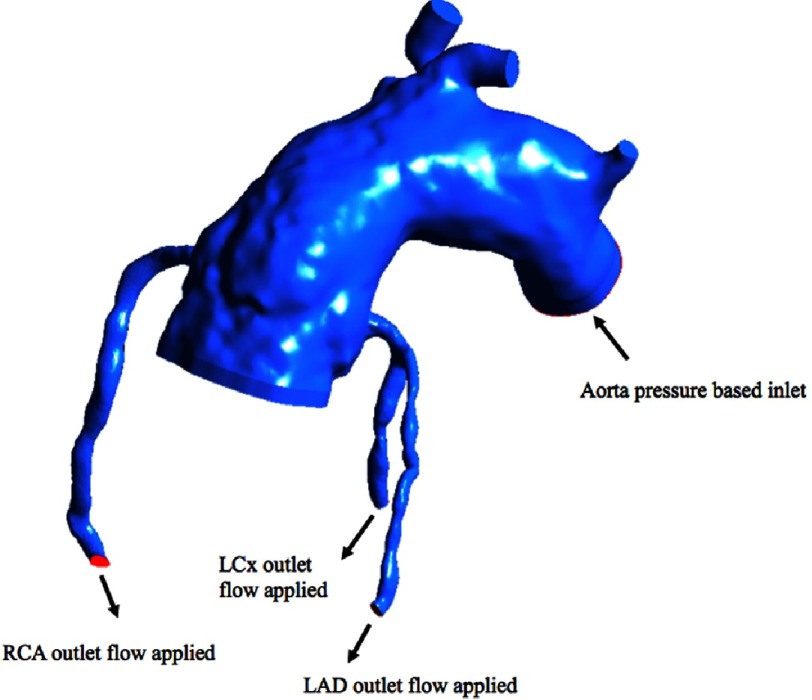
Modal evaluation in diastole.

**Figure 7A–B.**
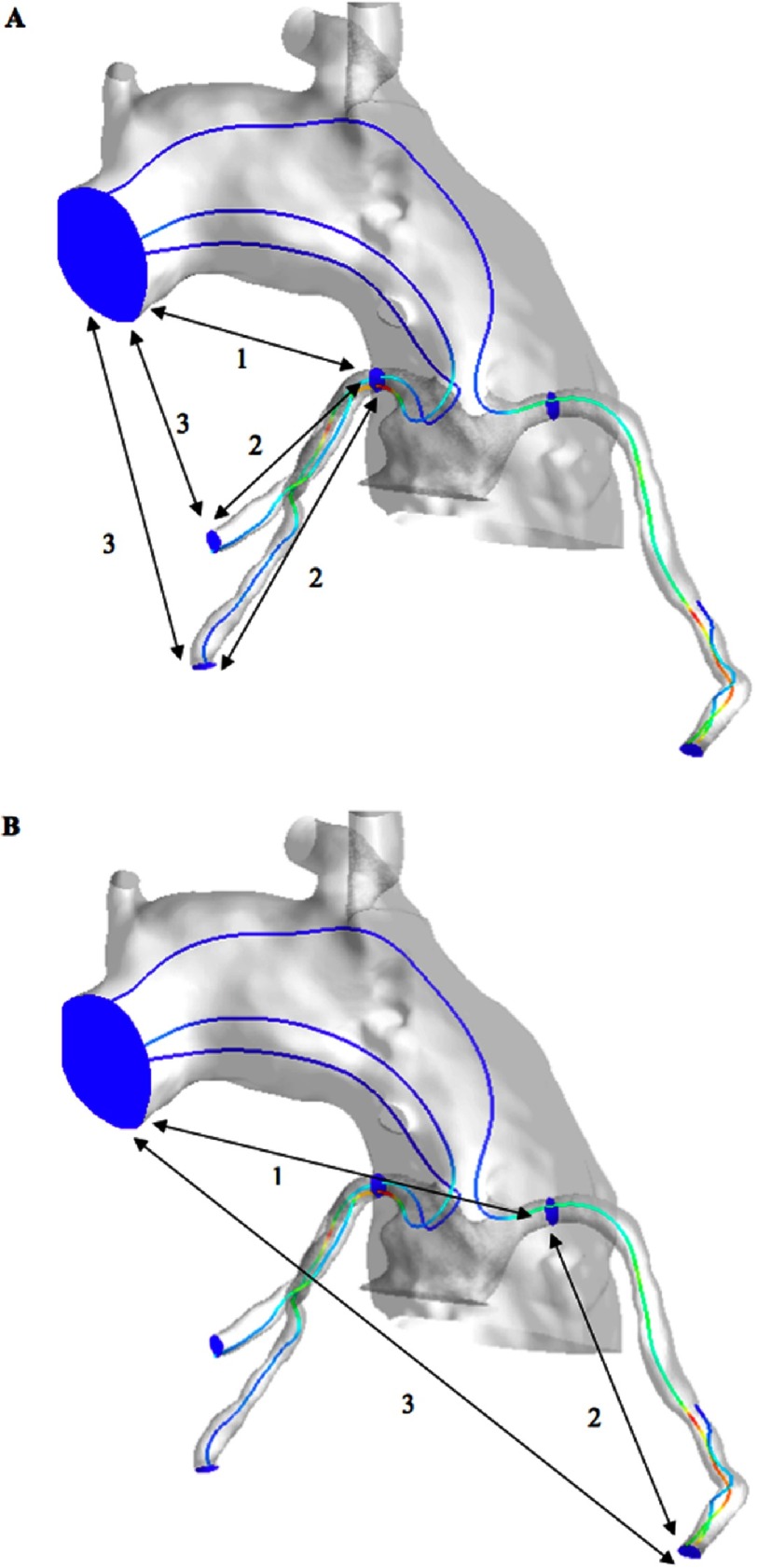
Pressure measurement locations in diastole. Total pressure drop was considered between the aortic inlet and distal left and distal right main planes (1), between distal main planes and the coronary outlets (2) and the cumulative drop from aortic inlet to coronary outlets (3) = (1) + (2).

### Modal evaluation: determining flow improvements from transferring the coronaries

Blood flow in the coronaries occurs throughout the heartbeat, during both systole and diastole but mostly during diastole. During systole blood enters the coronaries being pumped from the ventricle and being resisted by the impedance of the coronary vessels. To model this, we simulated each geometry (pre-transfer and post-transfer) with 3 different flow rates through the pulmonary root and the reconstructed aorta and monitored how much blood flows into the coronaries. We then compared the coronary flow rates between the two models (Figure 5).

During diastole, blood is drawn into the coronaries via a pressure gradient created as the ventricle expands. Since we do not know this pressure gradient, and it is likely to vary generating a variable flow rate throughout diastole, we instead applied 3 flow rates through the coronaries and then we measured the total pressure drop from the aortic inlet through the coronaries. A lower pressure drop is equivalent to more flow at a given pressure gradient (Figure 6).

In diastole, we determined the pressure drop in 3 locations as shown in Figure 7. These were between the aortic inlet and distal left and right main planes (1), between distal main planes to the coronary outlets (2), and the cumulative drop from aortic inlet to coronary outlets (3) = (1) + (2). We were more interested in the pressure drop between the aortic inlet and distal left main (just before its bifurcation) and between the aortic inlet and distal right main (a point at the proximal RCA that corresponds to the distal left main and we called it the distal right main), (No.1 in Figure 7), because any difference between the two models in this value will be as a result of the geometry difference between the two models i.e., the coronary transfer to the pulmonary root. The geometry of the coronaries themselves was similar between the two models.

### Target coronary artery average flow rates

We assumed the cardiac output (CO) and coronary splits that matched with the age of the patient we derived from the MRI.

CO = 2.5 L/min = 41.67 mL/s

• Left and right coronary arteries receive 2% each of the total flow = 41.67*2% = 0.833 mL/s
• Total RCA = 0.833 mL/s
  – Systole flow 15%= 0.833*15% = **0.125 mL/s**
  – Diastole flow 85% = 0.833*85% = **0.708 mL/s**
• Total LCx is 40% of left coronary flow = 0.833*40% = 0.333 mL/s
  – Systole flow 15% = 0.333*15% = **0.050 mL/s**
  – Diastole flow 85% = 0.333*85% = **0.283 mL/s**
• Total LAD is 60% of left coronary flow = 0.833*60% = 0.500 mL/s
  – Systole flow 15% = 0.5*15% = **0.075 mL/s**
  – Diastole flow 85% = 0.5*85% = **0.425 mL/s**

### Systole boundary conditions

To simulate the two periods of systole and diastole, we used two sets of boundary conditions. During systole, 3 fixed aortic flow rates were applied in 3 separate simulations to represent 3 conditions: a maximum flow at the onset of systole when flow is highest, a minimum flow at the end of systole when flow is lowest, and an average flow rate during the full systole period. A diastolic pressure and resistance was applied to the coronary arteries and aortic outlets. The resistance at each outlet is adjusted in the average inlet flow simulation until both target systolic pressure in the aorta (90 mmHg) is reached and the correct average flow rates are achieved through each coronary artery outlet. The same outlet boundary conditions were then used with the high (maximum) and low (minimum) flow rate through the aorta. The subsequent flow rates in the coronaries were measured.

During systole, flow into the aorta was a fixed flow rate:

Cardiac output (CO) = 2.5 L/min

Heart rate = 100 BPM

⇒ Flow = 25 mL/beat

Systole duration = 0.23 s

⇒ Average aortic flow rate during systole = 108.7 mL/s = 0.0001087 m^3^/s

Inlet area = 0.0004727 m^2^

⇒ Average inlet velocity = 0.23 m/s

**Figure 8.**
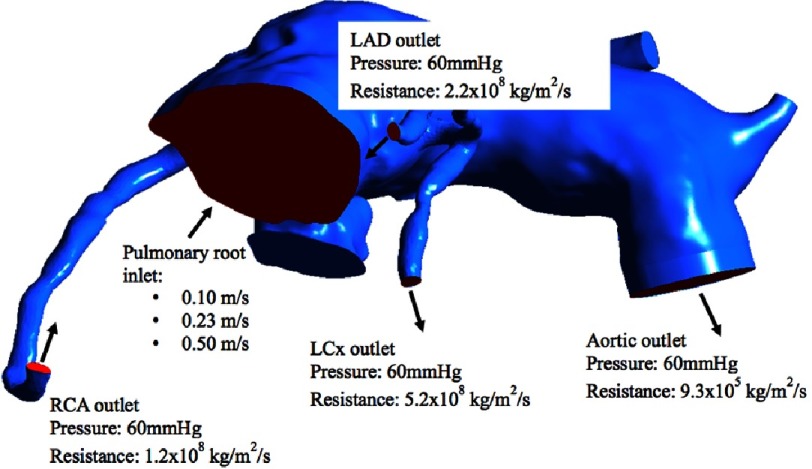
Systole boundary conditions with 3 flow rates through the aorta. Resistance across the outlet block creates an internal aortic pressure of 90 mmHg. The 60 mmHg pressure outer boundary condition represents far field pressure.

We know the aortic velocity is not constant during systole. There is a high velocity at onset and a slowing down during contraction. As such, 3 flow rates were applied to 3 separate simulations; 0.23 m/s (average), 0.50 m/s (maximum) & 0.10 m/s (minimum). The systolic boundary conditions are summarized in Figure 8.

### Diastole boundary conditions

**Figure 9.**
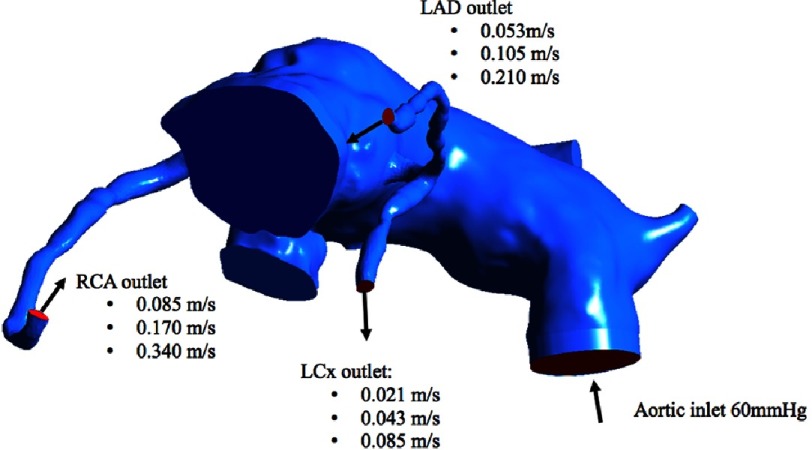
Diastole boundary conditions with 3 outlet velocities for 3 flow rates through the coronaries

During diastole, the pulmonary root inlet is set to a wall boundary condition to prevent flow across it. This represents the closed pulmonary (systemic) valve. Since we do not know the suction pressure created by myocardial relaxation, but we do know the coronary arteries flow rates, the flow rates were applied to the 3 coronary artery outlets, and the pressure drop from the aortic inlet to the coronary outlets was measured. A lower pressure drop is indicative of greater blood flow. A diastolic pressure boundary (8,000 Pa = 60 mmHg) was applied to the aortic outlet, which is now an inlet during the diastole phase, letting blood be drawn back into the aorta to supply the coronary arteries. As with the systolic simulations, 3 flow rates through the coronaries were simulated: average, maximum, and minimum. This represents different flow rates the coronaries will receive throughout diastole; maximum at onset, minimum at the end, and average during the full diastole period. The diastolic boundary conditions are summarized in Figure 9.

The same systolic and diastolic boundary conditions were used for both pre-transfer and post-transfer models. The principle is simple; for systolic flow (constant pressure gradient), we monitored coronary flow rate. A higher flow rate at a fixed pressure gradient is better. For diastolic flow (constant flow rate), we monitored the pressure drop. Lower drop is better. Comparing the results of the 2 models will tell us which will have a better hemodynamic coronary circulation.

### Other operating conditions

All other boundaries are adiabatic, smooth, no slip walls. The blood had a fixed density of 1025 kg/m^3^ and the viscosity was modelled with the Cross viscosity model using the following equation and applied with the User Defined Function (UDF):

\begin{eqnarray*}{\mu }_{cross}={\mu }_{Newtonian}+ \frac{({\mu }_{0}-{\mu }_{Newtonian})}{(1+{ \left( 1.007\times \gamma \right) }^{1.028})} \end{eqnarray*}

Where;

*μ*_*cross*_ = non-Newtonian viscosity (cps)

*μ*_0_ = 56.0 cps (viscosity at zero shear)

*μ*_*Newtonian*_ = 3.5 cps (Newtonian viscosity, infinite shear viscosity)

*γ* = shear strain rate (1/sec)

### Simulation summary

Tables 1 and 2 summarize our simulations. All 6 conditions were applied to both pre-transfer and post-transfer models, for a total of 12 simulations.

**Table 1:** Aortic flow rates during systole.

Systole	Aortic root inlet velocity (m/s)
Average flow	0.23
Minimum flow	0.10
Maximum flow	0.50

**Table 2:** Coronary flow rates during diastole.

Diastole	RCA outlet flow velocity (m/s)	LCx outlet flow velocity (m/s)	LAD outlet flow velocity (m/s)
Average flow	0.170	0.043	0.105
Minimum flow	0.085	0.021	0.053
Maximum flow	0.340	0.085	0.210


**Figure 10.**
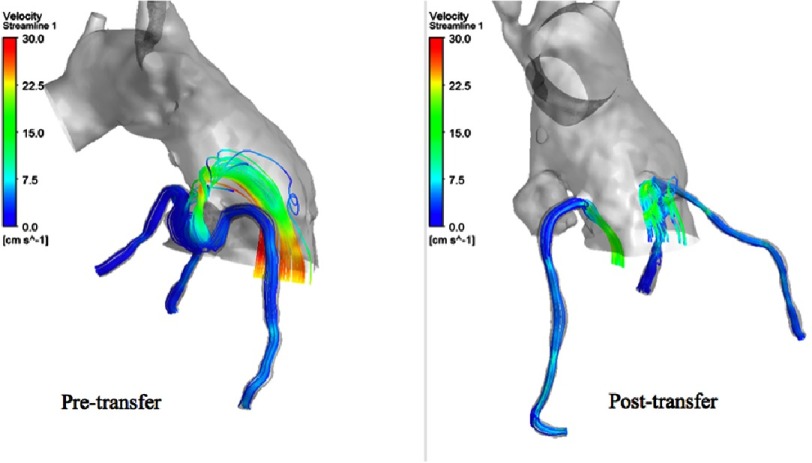
Streamlines through the coronaries during systole.

**Figure 11.**
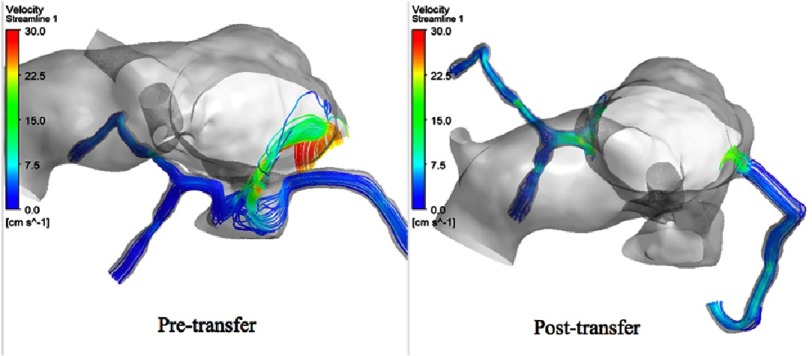
Streamlines through the coronaries during systole.

**Figure 12.**
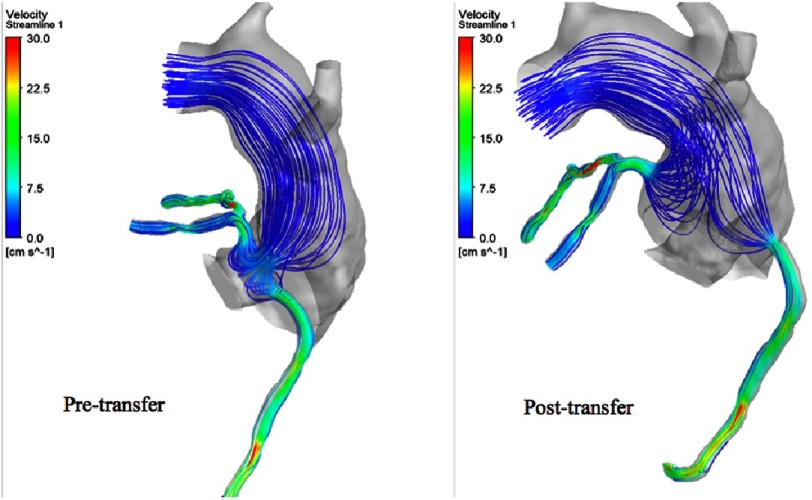
Streamlines through the coronaries during diastole.

**Figure 13.**
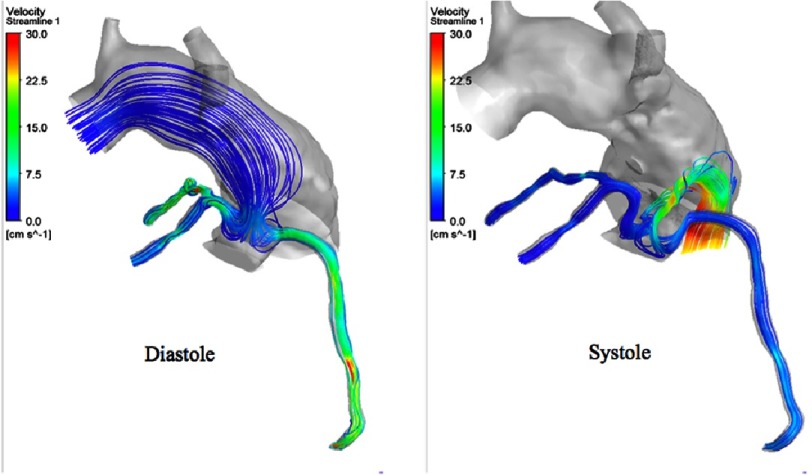
Streamlines through the coronaries during systole and diastole in the pre-transfer model.

**Figure 14.**
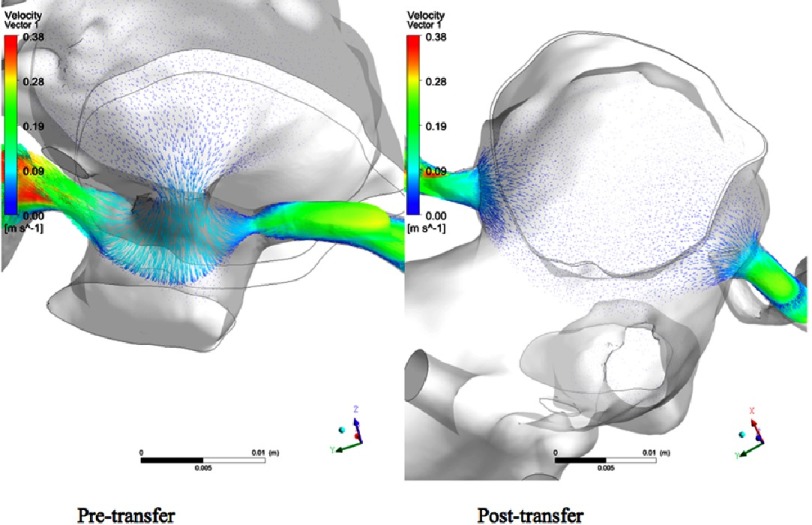
Velocity vectors of flow entering the coronaries in the pre- and post-transfer models during diastole at maximum flow rate.

**Figure 15.**
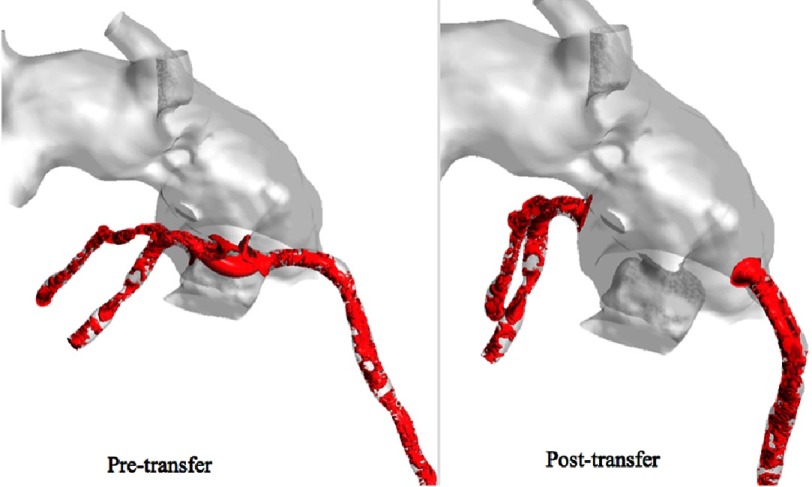
Isosurface of swirl strength during average diastolic flow.

**Figure 16.**
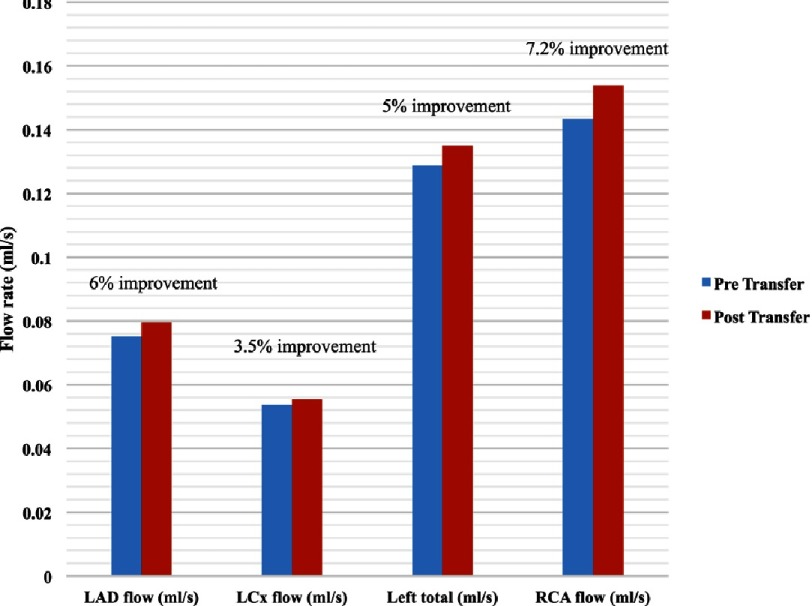
Coronary arteries flow rates during systole at average aortic flow.

## Results

### Streamlines through the coronaries

Figure 10 shows the flow streamlines during systole. As we can observe in the pre-transfer model, there is a retrograde flow pattern component towards the coronary orifices, and there is a very high velocity flow at the plane of the coronary buttons because of the hypoplastic aortic root and sinuses.

On the other hand, in the post-transfer model, the flow streamlines look more physiologic and have a full antegrade pattern, and without the high velocity flow at the planes of the coronary buttons, which is obviously related to their attachments to the well-developed pulmonary root. Figure 11 shows the flow streamlines during systole from a different view angle. Figure 12 shows the coronary flow patterns during diastole. The same observations are noted again in the pre-transfer model compared to the post-transfer model during diastole phase. Figure 13 shows the velocity streamlines during systole and diastole in the pre-transfer model. Velocity vectors are shown in Figure 14. A velocity vector has a direction and a magnitude (speed) of the flow. In the pre-transfer model, the plot of velocity vectors show arrows of direction towards the native hypoplastic aortic root first, then towards the coronary sinuses, and there is a high speed of flow through the native hypoplastic aortic root across the aortic sinuses. While in the post-transfer model, the vectors completely direct towards the coronary orifices in a full antegrade pattern, and without associated high velocity across the pulmonary root.

Figure 15 shows the isosurface of swirl strength during diastole. In the pre-transfer model, there is a measured swirl in the native hypoplastic aortic root, but there is none in the pulmonary root of the post-transfer model. Swirl flow is undesirable hemodynamic effect. It is always accompanied by an increase in velocity fluctuations, pressure drop, and flow reversal.

### Coronary artery flow rate during systole

At average aortic flow, the blood flow rate in all coronary branches was higher in the post-transfer model in the following proportions: left main 5% higher, LAD 6% higher, LCx 3.5% higher, RCA 7.2% higher flow rates. Figure 16 shows the flow distributions in both models at average aortic flow.

### Total pressure drop during systole

Because of different coronary systolic flow rates between both models, we cannot compare the total pressure drop directly, however it is worth noting the following: 1) total pressure drop through the system is dominated by drop in the coronaries themselves, and 2) across the pulmonary root inlet to the distal main of the left and right coronaries, for similar flow rates the total pressure drop was significantly lower in the post-transfer model.

### Total pressure drop during diastole

#### At average coronary flow

The total pressure drop between the aortic inlet and distal left main was 15.6% lower in the post-transfer model than pre-transfer model. The total pressure drop between the aortic inlet and distal right main was 36.2% lower in the post-transfer model than pre-transfer model.

#### At maximum coronary flow

The total pressure drop between the aortic inlet and distal left main was 22% lower in the post-transfer model than pre-transfer model. The total pressure drop between the aortic inlet and distal right main was 47.1% lower in the post-transfer model than pre-transfer model.

#### At minimum coronary flow

**Figure 17.**
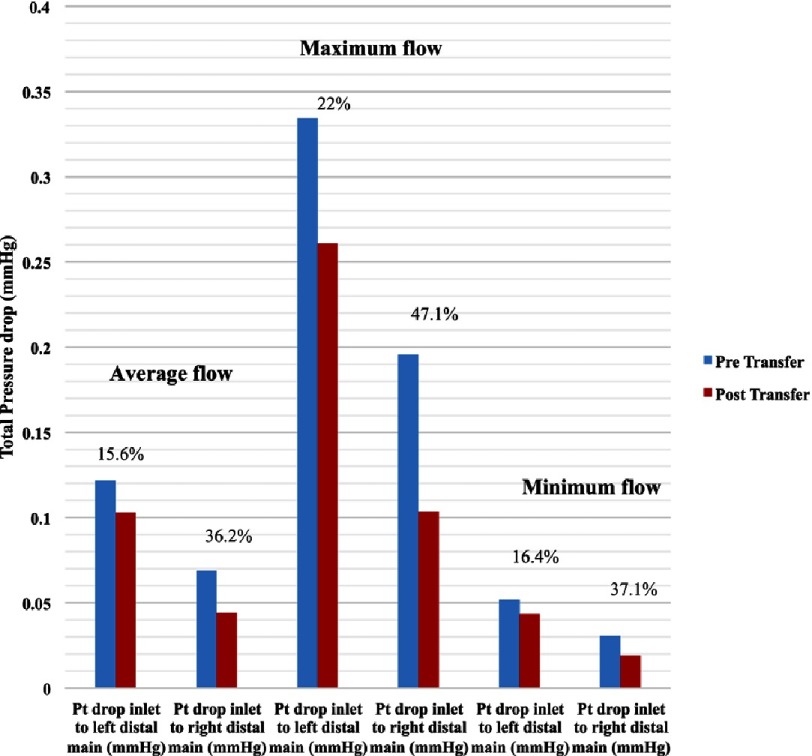
Total pressure drop from aortic inlet to distal main locations during diastole at all coronary flow rates.

The total pressure drop between the aortic inlet and distal left main was 16.4% lower in the post-transfer model than pre-transfer model. The total pressure drop between the aortic inlet and distal right main was 37.1% lower in the post-transfer model than pre-transfer model. Figure 17 shows the total pressure drop between the aortic inlet and distal left main and distal right main at the 3 coronary flow rates during diastole.

## Discussion

The Norwood procedure was introduced by Dr. Willian Norwood and colleagues in 1981.^3,4^

Bove and colleagues found better coronary hemodynamics after Sano shunt compared to BT shunt^5^. Our CFD simulation predicted more physiologic streamlines in the post-transfer model in both systolic and diastolic phases. Furthermore, the systolic flow rate has increased to all coronary branches when the left and right coronary buttons were moved to the well-developed pulmonary root for a range of flow velocities through the aorta particularly to the RCA. Although it was somehow not a substantial improvement in the systolic flow rate, our simulation involved only 15% of the coronary flow that occurs in systole. Most of the coronary flow simulation occurred in diastole (85%), and we found a large difference in diastole between the two models in favor of post-transfer in the form of total pressure drop, which will result in a better diastolic flow. Because of different flow rates between pre- and post-transfer, we cannot compare total pressure drop directly during systole, however it is worth noting that total pressure drop through the system is dominated by drop in the coronaries themselves. Across the pulmonary root inlet to the distal main of the left and right coronaries, for similar flow rates the total pressure drop was significantly lower in the post-transfer model. So, it seems reasonable to conclude that the reduction in total pressure drop from the pulmonary root inlet to the distal left and right main locations is the cause of increased flow in the individual coronaries.

With respect to coronary flow hemodynamics in diastole, our CFD simulation predicted that total pressure drop from the aortic inlet to the coronary outlets is dominated by drop in the coronaries themselves. For a given flow, total pressure drop within the coronaries themselves was similar in both pre- and post-transfer models, which we would expect since the flow and the geometry are the same. Total pressure drop from the aortic inlet to both distal left and right main locations decreased significantly in the post-transfer model for a range of flow velocities as shown above. This results in a net smaller total pressure drop in the post-transfer model through the whole system (from aortic inlet to coronary outlets, no. 3 in Figure 7) in all flow rates.

We believe that coronary transfer should be considered whenever the pulmonary root supplies the systemic circulation such as Norwood, DKS, and Yasui operations. However, we believe that the coronary circulation is less compromised post DKS compared to Norwood, and as such the coronary transfer after DKS may not produce the same degree of hemodynamic advantages as would it be produced after the Norwood procedure for the following reasons.

First, the coronaries after the Norwood arise from hypoplastic aortic root and sinuses and the sinuses have a major role in the physiology of coronary circulation. This is not always the case during DKS shunt in which the coronaries may arise from well-developed aortic root and sinuses.

Second, the cavo-pulmonary shunt could be performed in concomitant with DKS shunt. This has an advantage in eliminating the risk of coronary steal, and the circulation is in-series and not parallel compared to the balanced parallel circulation after DKS with aorto-pulmonary shunt.

Third, performing a palliative arterial switch operation with coronary transfer in the morphologic scenarios where DKS is an alternative is probably more physiologic, and has an advantage in preserving the geometry of the pulmonary valve.

After showing the benefits of coronary transfer to the pulmonary root even without simulating the systemic to pulmonary shunt in this study, simulating the systemic to pulmonary shunt will obviously demonstrate the hemodynamic advantages of the coronary transfer in a more significant degree.

## Limitations and future work

In this study, our goal was first to demonstrate the effect of coronary transfer to the pulmonary root on the coronary flow hemodynamics. So, we did not yet simulate the systemic to pulmonary shunt in this study for simplicity. We are currently simulating the BT shunt and Sano shunt in both pre- and post-transfer models. This will give us a total of four models to simulate and compare coronary flow patterns in between. This will be done to evaluate our fourth hypothesis i.e., minimal or no effect of the type of shunt (Sano vs. BT) on the coronary blood flow after performing the Norwood with our modification.

## Summary

There is a concern about the adequacy of myocardial perfusion after the Norwood procedure. Our proposed modification as proved by this CFD study will address and hopefully resolve this concern. This modification can be applied to Norwood, DKS, and Yasui operations where the pulmonary root supplies the systemic circulation in the setting of single or biventricular repair. In this study we proved our first three hypotheses. We are currently evaluating our fourth hypothesis.
